# Cellulose Fiber Rejects as Raw Material for Integrated Production of *Pleurotus* spp. Mushrooms and Activated Biochar for Removal of Emerging Pollutants from Aqueous Media

**DOI:** 10.1021/acsomega.2c06453

**Published:** 2023-01-31

**Authors:** Alejandro Grimm, Feng Chen, Glaydson Simões dos Reis, Van Minh Dinh, Santosh Govind Khokarale, Michael Finell, Jyri-Pekka Mikkola, Malin Hultberg, Guilherme L. Dotto, Shaojun Xiong

**Affiliations:** † Department of Forest Biomaterials and Technology, Swedish University of Agricultural Sciences, UmeåSE-901 83, Sweden; ‡ Technical Chemistry, Department of Chemistry, Chemical-Biological Centre, Umeå University, UmeåSE-901 87, Sweden; § Industrial Chemistry and Reaction Engineering, Johan Gadolin Process Chemistry Centre, Åbo Akademi University, Åbo-TurkuFI-20500, Finland; ∥ Department of Biosystems and Technology, Swedish University of Agricultural Sciences, AlnarpSE-230 53, Sweden; ⊥ Research Group on Adsorptive and Catalytic Process Engineering (ENGEPAC), Federal University of Santa Maria, Av. Roraima, 1000-7, RS, Santa MariaBR 97105-900, Brazil

## Abstract

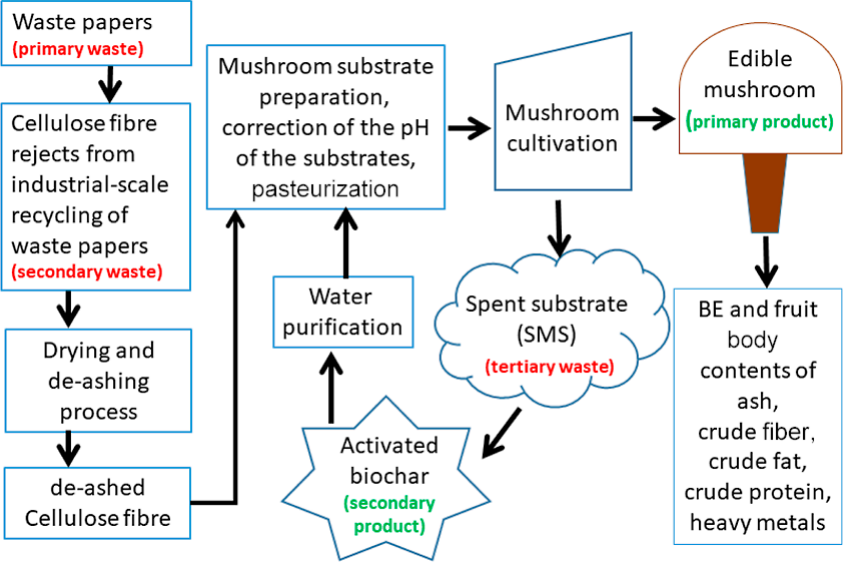

Cellulose fiber rejects from industrial-scale recycling of waste papers were dried and de-ashed using a combined cyclone-drying and sieving process. The upgraded fiber reject was used as a component of substrates for the cultivation of *Pleurotus ostreatus* and *Pleurotus eryngii* mushrooms. Acetic acid (AA) and acid whey (AW) were used to adjust the pH of fiber reject-based substrates. Spent substrate (SMS) was used for the production of activated biochar using H_3_PO_4_ and KOH as activating agents and pyrolysis temperatures of 500, 600, and 700 °C. The effectiveness of the biochars in removing pollutants from water was determined using acetaminophen and amoxicillin. By using a feeding rate of 250 kg/h and a drying air temperature of 70 °C, the moisture content of the raw fiber rejects (57.8 wt %) was reduced to 5.4 wt %, and the ash content (39.2 wt %) was reduced to 21.5 wt %. Substrates with 60 and 80 wt % de-ashed cellulose fiber were colonized faster than a birch wood-based control substrate. The adjustment of the pH of these two substrates to approximately 6.5 by using AA led to longer colonization times but biological efficiencies (BEs) that were higher or comparable to that of the control substrate. The contents of ash, crude fiber, crude fat, and crude protein of fruit bodies grown on fiber reject-based substrates were comparable to that of those grown on control substrates, and the contents of toxic heavy metals, that is, As, Pb, Cd, and Hg, were well below the up-limit values for food products set in EC regulations. Activated biochar produced from fiber reject-based SMS at a temperature of 700 °C resulted in a surface area (BET) of 396 m^2^/g (H_3_PO_4_-activated biochar) and 199 m^2^/g (KOH-activated biochar). For both activated biochars, the kinetics of adsorption of acetaminophen and amoxicillin were better described using the general order model. The isotherms of adsorption were better described by the Freundlich model (H_3_PO_4_-activated biochar) and the Langmuir model (KOH-activated biochar).

## 1 Introduction

According to statistics, the current global annual production of cardboard and paper totals approximately 400 Mt and the projected consumption in 2031 is 466 Mt.^1^ Paper plays an important role in our everyday activities; therefore, the recycling of used paper has increased significantly during the past decade to conserve natural resources and energy.^2^ Every time paper is recycled, a waste known as cellulose fiber rejects is generated. This waste can be split into different types,^3^ from worthless coarse objects to low-quality cellulose fiber that can be considered an interesting material for growing edible white-rot fungi. According to statistics, the total world production of edible mushrooms in 2020 was approximately 43 Mt, with China being the largest producer with approximately 40 Mt.^4^ Commercial white-rot fungi such as *pleurotus* spp. are commonly cultivated on substrates made of diverse types of lignocellulosic biomasses such as cereal straw,^5^ wood sawdust,^6^ corncobs and sugarcane bagasse,^7^ and cottonseed hull,^8^ among others. Cultivation of this type of mushroom generates significant quantities of spent substrate (SMS) that the industry struggles to dispose of. Singh *et al.*(9) reported that approximately 300–400 kg of SMS is left for every 100 kg of mushroom produced, and an average mushroom farm discards about 24 tons of SMS per month. Due to the ability of this type of fungi to degrade lignin, cellulose, and hemicellulose, some mushroom industries use white-rot fungi SMS in substrates for fungi that grow on compost.^10^ However, this practice is not always economically viable or resolves completely the disposal problem, meaning that considerable quantities of SMS are landfilled, used as fuel for heating purposes, or burned in the field.

Since significant parts of the population in the world have limited access to clean water supplies,^11^ and the production and processing of foodstuffs require considerable quantities of water of appropriate quality, the development of methods to convert inexpensive biomass waste such as SMS into added-value materials such as activated biochar for treatment of water may lead to huge economic benefits.^12^ Water treatment methods based on carbon-based adsorbents are well known for their high efficiency in the removal of organic and inorganic pollutants from water. Few research studies have been devoted to evaluating SMS’s suitability for producing carbon-based adsorbents. Published research includes the treatment of dye-laden wastewater,^13^ sulfide adsorption,^14^ supercapacitors,^15^ and adsorption of toxins such as microcystin^16^ or drugs such as acetaminophen.^17^ None of these works studied the suitability of cellulose-rich waste generated during industrial-scale recycling of waste papers for the combined production of edible mushrooms and carbon-based adsorbents for water treatment.

Through a circular economy concept, the objective of this research was to: (a) evaluate the possibility of reducing the amount of contaminants such as ash and microplastics contained in cellulose fiber rejects (*secondary waste*) generated during industrial-scale recycling/processing of waste papers (*primary waste*) by means of a combined drying and de-ashing process; (b) evaluate the suitability of the upgraded fiber reject for its use in substrates for the cultivation of *Pleurotus* spp. mushrooms (*primary product*); (c) evaluate the effect that the correction of the pH of the fiber reject-based substrates has on the biological efficiency (BE) and fruit bodies’ quality parameters and; (d) evaluate the possibility of reusing SMS (*tertiary waste*) as raw material for the production of activated biochar (*secondary product*).

## 2 Materials and Methods

### 2.1 Raw Materials Used for the Production of Mushroom Substrates

The ingredients used for the production of substrates include (a) light coarse cellulose fiber rejects collected from the Swedish Cellulose Company (SCA) Obbola, Umeå, Sweden; (b) birch (*Betula* ssp.) wood sawdust; (c) wheat bran and barley grain (Lantmännen Lantbruk, Sweden) were used to promote productivity; and (d) calcium carbonate (Nordkalk Oy AB, Sweden), acetic acid (AA) 24% aqueous solution (Perstorp AB, Sweden), and sweet-whey powder (Norrmejerier, Umeå, Sweden) from the production of cheese were used to adjust the pH of the substrates. The whey powder was reconstituted in water at 50 g/L. The solution was inoculated with *Lactobacillus plantarum* strain LB14, thoroughly mixed and fermented at 37 °C until pH 4 to obtain acid whey (AW).

### 2.2 Pre-Processing of Raw Cellulose Fiber Rejects—Drying and De-Ashing

**Figure 1:**
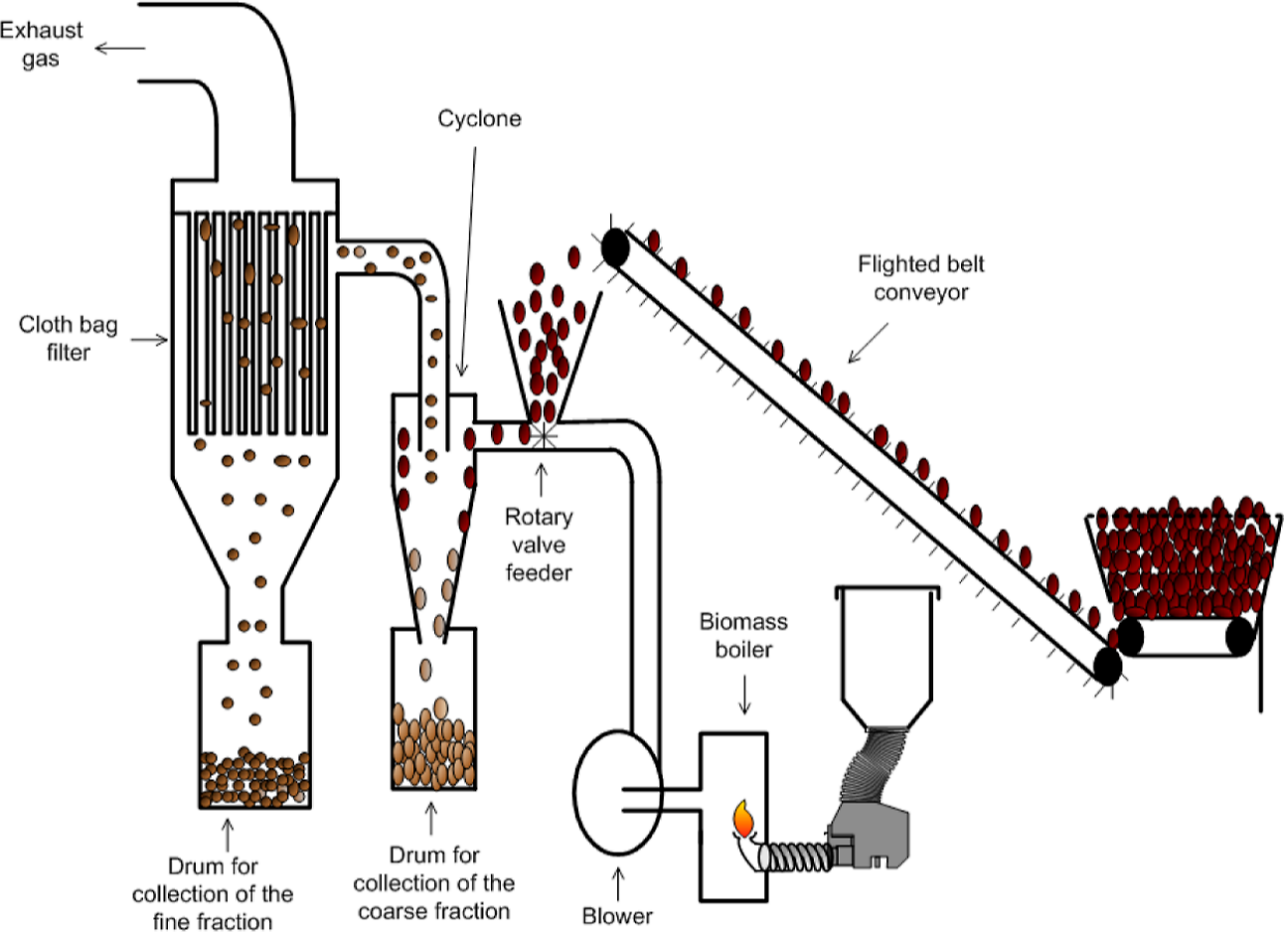
Schematic overview of the pilot-scale cyclone dryer experimental setup (not to scale).

The fiber rejects used in this work (200 kg, moisture content of approximately 58 wt % w.w.) was composed mainly of cellulose fiber and minor amounts of contaminants such as microplastics and ash, that is, calcium carbonate, kaolin, and sand, among others. The material was processed using a pilot-scale cyclone dryer that allows for combined drying and separation of feedstock components according to their particle size.^18^Figure 1 shows a schematic overview of the experimental setup used in this work, and Supporting Figure S1 shows a photograph of the installation.

The dryer was operated at a feeding rate of 250 kg/h and a drying air temperature of 70 °C. Air was drawn in by an industrial air blower (150 kW) and heated up with the aid of a biomass boiler (Säätötuli Oy) equipped with a 50 kW pellet burner and an integrated air-cooled heat exchanger. The power output of the pellet burner was adjusted to obtain the desired process air temperature. Hot air from the blower outlet port was fed into the cyclone.

The feeding system consists of a hopper and a belt conveyor resting on weighing pads connected to a digital indicator. The speed of the belt conveyor was adjusted to regulate the feeding rate. The fiber reject was transported from ground level to the cyclone’s rotary valve feeder using a flighted belt conveyor. Once the feedstock enters the cyclone, the force of the hot-air stream leads to a disaggregation due to particle–particle and particle-cyclone wall collisions, which helps increase the drying rate. From the process, two fractions were obtained, one that flowed out of the cyclone-cone outlet into a drum below (here called, coarse fraction), and another formed by uncollected dust (here called, fine fraction) that flowed out of the cyclone gas outlet duct into a cloth bag filter. The coarse fraction obtained from the cyclone dryer was sieved using a pilot-scale screening device (Mogensen AB, Sweden) equipped with sieves of 8 and 4 mm to eliminate fine cellulose dust and remnants of contaminants such as microplastics. A fraction with a particle size of ≥8 mm, which consisted mainly of lumps of cellulose fiber [here called, de-ashed fiber reject (DFR)], was chosen as raw material for the preparation of the mushroom substrates.

### 2.3 Substrate Compositions

The formulation of the tested substrates is shown in Table 1. Two substrates were produced with 60 and 80 wt % de-ashed fiber rejects (DFR-60 and DFR-80). A standard birch sawdust substrate formula (DFR-00) was used as a comparison. The mixtures were made using a ribbon mixer, and the moisture content was adjusted to 65 wt % by adding water. The pH of each substrate was measured with a Testo 206 pH2 device (Testo SE & Co. KGaA, Germany). The pH of the DFR-00 substrate was lower than the optimal pH for mycelium growth (∼6.5) and, therefore, was corrected by adding CaCO_3_. The pH of the substrates DFR-60 (7.10) and DFR-80 (7.73) was slightly high; therefore, additional substrates of these two were prepared by adding AA or AW together with water to obtain mixtures with a moisture content of 65 wt % and a pH of approximately 6.5. 10 blocks of 1 kg were produced for each type of substrate using polypropylene breathing bags (SacO_2_, Microsac, USA).

**Table 1:** Mushroom Substrate Formulations

	components of the initial substrate (g/kg substrate)								
substrate ID^a^	de-ashed fiber reject	birch sawdust	wheat bran	barley grain	CaCO_3_	acetic acid (24%)	acid whey (50 g/L)	water	pH^b^
DFR-00	0	280	35	35	3	0	0	650	6.51
DFR-60	210	70	35	35	0	0	0	650	7.10
DFR-80	280	0	35	35	0	0	0	650	7.73
DFR-60 + AA	210	70	35	35	0	13	0	637	6.52
DFR-80 + AA	280	0	35	35	0	22.75	0	627.25	6.54
DFR-60 + AW	210	70	35	35	0	0	390	260	6.49
DFR-80 + AW	280	0	35	35	0	0	650	0	6.56

a Numbers in the substrate ID denote the amount of de-ashed fiber reject in wt % dry mass.

b Average value of three measurements.

### 2.4 Characterization of the Substrates

Representative samples of each substrate were dried at 105 °C to constant weight and hammer milled. The ash content of each substrate was determined according to the ISO 18122:2015a standard method. The elemental analysis of the substrates (Table 2) was carried out by a certified laboratory (Eurofins, Sweden) according to standard methods. The analysis of the substrates with AA and AW is not shown because these additives do not contribute substantially to the final elemental composition. Only the AW increased the nitrogen content slightly, that is, 0.85 wt % dw. (DFR-60 + AW) and 1.02 wt % dw. (DFR-80 + AW), but the other elements remained at the same level as that of the DFR-60 and DFR-80 substrates.

**Table 2:** Characterization of the Raw Materials and Mushroom Substrates

		raw fiber reject	de-ashed fiber rejects (DFR)	wheat bran	barley grain	birch sawdust	DFR-00	DFR-60	DFR-80
moisture content	wt % w.w.	57.8	5.4	2.7	3.3	4.1	65.8	66.1	65.9
ash content	wt % d.w.	39.2	21.5	6.7	2.3	0.3	1.1	13.9	18.1
sulfur (S)	wt % d.w.	0.1	0.1	0.2	0.1	0.0	0.0	0.1	0.1
chlorine (Cl)	wt % d.w.	0.2	0.1	0.1	0.2	0.0	0.0	0.1	0.1
carbon (C)	wt % d.w.	33.6	39.8	46.4	45.8	49.6	48.9	43.0	41.1
hydrogen (H)	wt % d.w.	3.9	4.7	6.0	6.0	6.1	6.1	5.2	5.0
nitrogen (N)	wt % d.w.	0.3	0.3	2.6	2.3	0.1	0.6	0.7	0.7
oxygen (O), calc.	wt % d.w.	22.7	33.5	38.0	43.0	43.9	43.2	37.0	34.9
aluminum (Al)	mg/kg d.w.	8600.0	6200.0	20.0	12.0	11.0	12.0	3725.4	4963.2
antimony (Sb)	mg/kg d.w.	1.4	0.4	0.5	0.5	0.4	0.4	0.4	0.4
arsenic (As)	mg/kg d.w.	1.0	0.6	0.1	0.1	0.1	0.1	0.4	0.5
barium (Ba)	mg/kg d.w.	210.0	60.0	10.0	1.6	21.0	18.0	41.4	49.2
beryllium (Be)	mg/kg d.w.	0.1	0.1	0.1	0.1	0.1	0.1	0.1	0.1
lead (Pb)	mg/kg d.w.	38.0	20.0	0.1	0.0	0.3	0.2	12.1	16.0
boron (B)	mg/kg d.w.	51.0	18.0	12.0	12.0	11.0	11.2	15.4	16.8
phosphorus (P)	mg/kg d.w.	180.0	150.0	9600.0	2900.0	380.0	1554.0	1416.0	1370.0
iron (Fe)	mg/kg d.w.	4800.0	2800.0	150.0	43.0	6.6	24.6	1700.6	2259.3
cadmium (Cd)	mg/kg d.w.	1.5	0.5	0.1	0.1	0.1	0.1	0.3	0.4
calcium (Ca)	mg/kg d.w.	92000.0	62000.0	1200.0	400.0	650.0	1880.0	37490.0	49760.0
potassium (K)	mg/kg d.w.	390.0	340.0	15000.0	5000.0	540.0	2432.0	2312.0	2272.0
cobalt (Co)	mg/kg d.w.	8.6	3.1	0.0	0.0	0.1	0.1	1.9	2.5
copper (Cu)	mg/kg d.w.	70.0	52.0	11.0	3.8	1.1	2.4	32.9	43.1
chromium (Cr)	mg/kg d.w.	14.0	0.2	0.2	0.1	0.3	0.3	0.2	0.2
mercury (Hg)	mg/kg d.w.	0.3	0.2	0.0	0.0	0.0	0.0	0.1	0.2
magnesium (Mg)	mg/kg d.w.	2500.0	2000.0	4400.0	1300.0	160.0	698.0	1802.0	2170.0
manganese (Mn)	mg/kg d.w.	140.0	98.0	86.0	9.9	44.0	44.8	77.2	88.0
molybdenum (Mo)	mg/kg d.w.	37.0	1.2	1.1	0.6	0.1	0.3	0.9	1.1
sodium (Na)	mg/kg d.w.	810.0	100.0	71.0	85.0	54.0	58.8	86.4	95.6
nickel (Ni)	mg/kg d.w.	310.0	11.0	0.6	0.3	0.3	0.3	6.7	8.9
tin (Sn)	mg/kg d.w.	12.0	4.9	0.1	0.1	0.1	0.1	3.0	3.9
titanium (Ti)	mg/kg d.w.	85.0	52.0	12.0	12.0	11.0	11.2	35.8	44.0
vanadium (V)	mg/kg d.w.	10.0	3.7	0.1	0.1	0.1	0.1	2.3	3.0
zinc (Zn)	mg/kg d.w.	190.0	130.0	82.0	23.0	22.0	28.1	92.9	114.5
silicium (Si)	mg/kg d.w.	32000.0	17000.0	590.0	2200.0	540.0	711.0	10587.0	13879.0

### 2.5 Substrate Pasteurization Treatment

The pasteurization of the substrate blocks was carried out in an oven at 85 °C for 4 h. This was enough to deactivate adventitious organisms and pathogens. Detailed information about the method can be found in previous studies.^19,20^

### 2.6 Inoculation and Incubation of the Substrates

Grain spawn of two species of *Pleurotus* mushroom was used for inoculation. For each type of the substrate, five blocks were inoculated with *Pleurotus ostreatus* (*P. ostreatus*) (strain M2140) and five blocks with *Pleurotus eryngii* (*P. eryngii*) (strain M2603) using a wet mass weight ratio of 2.5%. The inoculation was carried out in a laminar flow cabinet under sterile conditions. The pasteurized substrate bags were open, and the inoculum was placed on top of each substrate block. Thereafter, the bags were resealed with a plastic clip. The inoculated blocks were kept in a dark room at 22 °C and 70% RH until colonization was completed. To induce the fruiting phase, fully colonized blocks were cold shocked at 10 °C for 12 h and; thereafter, moved to a climate chamber where light (∼500 lx), air temperature (18 °C), and relative air humidity (90%) were controlled.

### 2.7 Mycelium Growth Rate, Biological Efficiency, and Substrate Degradation

The time required for the hyphae to cover 50 and 100% of the substrate block lateral surface was used to estimate the mycelium growth rate. These times were recorded manually for each substrate block taking the inoculation day as time zero. The BE was calculated according to eq 1
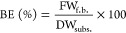
1where BE is expressed in percent, FW_f.b._ is the fresh weight of the fruit bodies (g) normalized to 90% moisture content (w.b.), and DW_subs._ is the dry weight (g) of the substrate block before inoculation.

The amount of the substrate degraded by the fungi was measured for each substrate block by the difference between the dry mass (determined by drying at 105 °C overnight) of the substrate before inoculation and after the harvest of the second flush of fruit bodies.

### 2.8 Chemical Analysis of the Mushroom Fruit Bodies

Three representative samples of each type of mushroom were collected from different blocks of each type of substrates. The moisture content of each sample was determined by freeze-drying using a CHRIST Alpha1-4 LSC plus device operated at −50 °C and 8 hPa. The dried samples were ground using a laboratory-scale hammer mill and used for chemical analysis. The contents of ash and fat were determined according to the procedures of the Association of Official Analytical Chemists.^21^ The total nitrogen content, determined using an elemental analyzer (Flash 2000, Thermo Fisher Scientific, Bremen, Germany), was used to estimate the crude protein content (N × 4.38). The crude fiber content was determined according to the method of Jennische and Larsson.^22^ The contents of potentially toxic heavy metals commonly found in foodstuffs [arsenic (As), lead (Pb), cadmium (Cd), and mercury (Hg)] were analyzed at a certified laboratory (Eurofins Food *&* Feed Testing Sweden AB) according to the SS-EN ISO 17294-2:2016/SS-EN 13805:2014 and the SS-EN 16277:2012 standard methods.

### 2.9 Statistical Analysis of the Results from Mushroom Cultivation

Cultivation experiments and related measurements were carried out at least in triplicate. IBM SPSS Statistics 27 software was used to evaluate the results using Tukey’s multiple comparison test with *p* < 0.05 as a threshold for statistical significance.

### 2.10 Production of Activated Biochar from Cellulose Fiber Reject Spent Substrates

The cellulose fiber reject-based SMS that led to the highest BE was used to produce activated biochar. Blocks of SMS were ground in a hammer mill equipped with a 2 mm sieve and used for the experiments.

SMS samples (50 g) were impregnated with a 8.6 M aqueous solution of phosphoric acid (H_3_PO_4_) or potassium hydroxide (KOH) using a weight ratio of 1 precursor: 3 acid/or base and allowed to soak for 12 h at 25 °C. The impregnated samples were pyrolyzed in a nitrogen gas (1 L/min) atmosphere using a tubular fixed-bed reactor heated externally with the aid of a muffle furnace. The temperature was raised from room temperature to the final treatment temperature (500, 600, and 700 °C, respectively) at a rate of 10 °C/min and held for 1 h. Thereafter, the reactor was allowed to cool while maintaining the N_2_ gas flow. The chars were washed several times with hot water until neutral pH and finally dried overnight in an oven at 105 °C to obtain the activated biochars.

### 2.11 Characterization of the Activated Biochars

Textural properties were examined using a sorptometer (Tristar 3000, Micromeritics Instrument Corp., Norcross, GA, USA) following standard procedures. The specific surface area was obtained according to the Brunauer–Emmett–Teller (BET) principle and pore size distribution using the Barrett–Joyner–Halenda (BJH) model.

Raman spectra of the activated biochars were obtained using a Bruker Bravo spectrometer (Bruker, Ettlingen, Germany). The samples were scanned in the 900–1800 cm^–1^ spectral range at 4 cm^–1^ resolution for 256 scans. The spectra were processed by smoothing (13 points) and vector normalization using OPUS software (version 7, Bruker Optik GmbH, Ettlingen, Germany) built-in functions.

### 2.12 Adsorption of Model Organic Micropollutants

Acetaminophen and amoxicillin were used as model organic micropollutants. The effect of the adsorbent dosage was performed by contacting 0.02 to 0.1 g of each activated biochar with 20 mL of each drug solution (200 mg/L) in 50 mL Falcon flat tubes. The solutions containing the adsorbent were agitated using an orbital shaker (IKA KS250, Labortechnik) at room temperature (22 °C) for 6 h. Next, the adsorbents were separated from the solutions by centrifugation (5000 rpm), and the residual concentration of acetaminophen or amoxicillin was determined by UV–vis spectrophotometry using a Shimadzu 1800 device at a λ_max_ of 243 and 228 nm, respectively.

The effect of the pH was determined by contacting 2 g/L each carbon with drug solutions of 200 mg/L with a pH from 3 to 10 that was adjusted by using HCl or NaOH. The solutions were agitated for 6 h; thereafter, the carbons were separated from the solutions, and the drug concentration decay was measured, as already described.

Kinetic measurements were carried out using an adsorbent dosage of 2 g/L and drug solutions with a concentration of 200 mg/L. The experimental conditions were maintained, as described above. Samples were periodically withdrawn, and the drug concentration decay was measured, as already described.

Equilibrium isotherms were obtained by contacting each activated biochar (2 g/L) with a range of drug solutions with an initial concentration between 10 and 1000 mg/L. The samples were agitated for the time required to attain equilibrium, and the drug concentration decay was measured, as already described.

The percentage of removal (eq 2) and the amount of drug uptake at equilibrium per unit of mass of activated biochar (eq 3) were used to quantify the amount of drug adsorbed by the biochars.
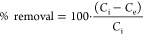
2
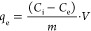
3where *C*_i_ is the initial concentration of the drug in the solution (mg/L), *C*_e_ is the equilibrium concentration of the drug in the solution (mg/L), *q*_e_ is the drug adsorption capacity at equilibrium (mg drug/g of the adsorbent), *V* is the volume of adsorbate solution (L), and *m* is the mass of the adsorbent (g).

## 3 Results and Discussion

### 3.1 Drying and De-Ashing of Cellulose Fiber Rejects

A mass balance showed that approximately 70 wt % dry mass of the cellulose fiber reject that was processed flowed out of the cone of the cyclone (coarse fraction), and the remaining part was collected in the cyclone cloth bag filter (fine fraction). The moisture content of the coarse and fine fractions was 5.4 and 1.2 wt %, respectively. The ash content of the fine fraction (approximately 80 wt %) made it unsuitable for further use and was discarded. The coarse fraction was sieved to obtain a material suitable for use in the mushroom substrates. Approximately 60 wt % dry mass of the raw cellulose fiber reject was left after the drying and sieving process [here called, de-ashed fiber reject (DFR)]. The ash content was reduced from 39.2 to 21.5 wt % (Table 2). Consequently, the concentration of potentially toxic heavy metals was considerably reduced, meaning that the ash removal process was capable of improving the quality of the cellulose fiber reject to a great extent.

### 3.2 Mycelium Growth, Harvest Time, Biological Efficiency, and Substrate Degradation

*P. ostreatus* and *P. eryngii* mycelia grew well on the studied substrates. Comparing the full colonization times between substrates (Table 3), one can see that both fungi species colonized the DFR-60 and DFR-80 substrates slightly faster than the control substrate (DFR-00). A reason for this could be the differences in particle sizes between substrates components, that is, DFR is composed of very small cellulose fibers that can be colonized faster than wood particles. Another reason for the faster colonization rates could be the differences in the lignin contents of the substrate components, that is, cellulose fiber, wood, wheat bran, and barley grain, as well as the presence of certain micronutrients in the substrate. The fungi cultivated in this work (white-rot) degrade the substrate through enzyme production. The copper (Cu) content in the DFR substrates (Table 2) is higher than that in the control substrate. Cu plays an important role in laccase production,^23^ that is, induces this lignin-degrading enzyme, which may lead to an increased lignin degradation rate, and thereby, become available for mycelium growth faster compared to the control substrate.

**Table 3:** Substrate Colonization and Harvest Times of *P. ostreatus* and *P. eryngii*

	colonization^a^		harvest^a^	
	50%	100%	1st flush	2nd flush
P. ostreatus				
DFR-00	11.2 ± 0.5^d^	25.7 ± 0.3^c^	29.7 ± 0.7^d^	52.8 ± 3.2^bc^
DFR-60	13.8 ± 0.5^c^	22.8 ± 0.3^d^	33.0 ± 0.1^cd^	51.5 ± 1.2^cd^
DFR-80	13.6 ± 0.2^c^	20.9 ± 0.4^e^	30.4 ± 1.4^cd^	43.7 ± 2.1^d^
DFR-60 + AA	16.5 ± 0.4^b^	32.2 ± 0.5^b^	40.8 ± 0.7^b^	60.5 ± 1.5^b^
DFR-80 + AA	21.8 ± 0.7^a^	42.3 ± 1.0^a^	51.2 ± 1.9^a^	70.5 ± 2.9^a^
DFR-60 + AW	13.7 ± 0.4^c^	23.2 ± 0.5^d^	32.0 ± 0.8^cd^	49.5 ± 2.1^cd^
DFR-80 + AW	15.8 ± 0.3^d^	23.5 ± 0.4^d^	34.3 ± 0.3^c^	48.8 ± 1.1^cd^
P. eryngii				
DFR-00	16.8 ± 0.4^b^	42.0 ± 0.3^c^	53.4 ± 1.6^c^	73.0 ± 0.1^e^
DFR-60	16.6 ± 0.4^b^	37.8 ± 0.4^de^	68.0 ± 3.9^b^	80.6 ± 2.1^cde^
DFR-80	16.8 ± 0.3^b^	35.8 ± 0.5^e^	69.3 ± 3.7^b^	89.3 ± 3.6^b^
DFR-60 + AA	21.4 ± 0.2^b^	47.2 ± 0.6^b^	73.4 ± 1.1^b^	88.2 ± 1.2^bc^
DFR-80 + AA	23.8 ± 2.1^a^	52.0 ± 1.3^a^	86.0 ± 1.9^a^	97.2 ± 2.5^a^
DFR-60 + AW	21.8 ± 0.4^a^	39.6 ± 0.2^cd^	68.4 ± 1.3^b^	81.8 ± 2.1^bcd^
DFR-80 + AW	17.3 ± 0.3^b^	36.0 ± 0.4^e^	63.5 ± 1.5^b^	77.3 ± 1.4^de^

a Mean values (days from substrate inoculation) with standard error, *n* = 5. Lack of letters in common indicates statistically significant differences (Tukey’s *t* test, *p* < 0.05) for comparisons of mean values between substrates.

For both fungi species, the full colonization of the DFR-60 + AW and DFR-80 + AW substrates was achieved slightly faster than that in the control (DFR-00) and the differences with the corresponding substrates without the addition of AW, that is, DFR-60 and DFR-80 were small (Table 3).

The addition of AA (DFR-80 + AA and DRF-60 + AA) led to significantly longer full colonization times for both mushroom species (Table 3). It was observed that the mycelium density in these substrates was much higher than that in the other substrates, that is, the mycelium grew slower but better. Probably, AA acted as a carbon source for fungi that can be easily consumed.^24^ Additionally, AA is secreted by white-rot fungi during the colonization of the substrate when sugars are broken down.^25,26^ The addition of AA probably had a synergistic effect on the degradation (hydrolysis) of the substrate, which led to a longer colonization time and as result a higher mycelium density.

The harvest times of the first and second flush of fruit bodies are shown in Table 3. AA delayed the harvest of the first flush of fruit bodies of both mushroom species, which is probably due to the slower colonization rate or longer time required for the mycelium to mature. In general, for the other substrates containing fiber reject, the time for the harvest of the first flush of *P. ostreatus* was comparable to that of the control substrate, and for *P. eryngii* took between 10 and 16 days more than that of the control substrate. Depending on the substrate, the second flush of fruit bodies of both species was harvested between 13 and 20 days after the first flush.

The BE of the 1st and 2nd flush of fruit bodies from the studied substrates is shown in Table 4. The DFR-80 + AA substrate led to the highest BE of the first flush of *P. ostreatus* with 57.7% compared to 43.5% for the control substrate. For the other substrates, the BE of the first flush was comparable to that of the control substrate with no significant differences. The highest BE of the second flush of *P. ostreatus* from substrates containing fiber rejects was for the DFR-80 + AW with 27.8% and DFR-80 with 24.8%. For the other substrates, the BE of the second flush ranged from approximately 14 to 20% compared to 16.5% for the control substrate. The substrates that led to the highest total BE of *P. ostreatus* were DFR-80 + AA (71.6%) and DFR80 + AW (68.8%) compared to 60.0% for the control substrate (DFR-00).

**Table 4:** Biological Efficiency of the First and Second Flush of *P. ostreatus* and *P. eryngii* and Amount of the Substrate Left after Cultivation

	BE (%)^a^			
	1st flush	2nd flush	total	amount of substrate left (wt % d.w.)^b^
P. ostreatus				
DFR-00	43.5 ± 1.4^b^	16.5 ± 2.5^bc^	60.0 ± 3.4^abc^	75.5 ± 0.4^a^
DFR-60	37.2 ± 2.3^b^	17.0 ± 0.8^bc^	54.2 ± 2.3^c^	65.8 ± 0.5^b^
DFR-80	37.8 ± 2.3^b^	24.8 ± 1.6^ab^	62.6 ± 0.7^abc^	62.6 ± 0.7^cd^
DFR-60 + AA	43.1 ± 3.7^b^	19.7 ± 2.6^abc^	62.7 ± 2.5^abc^	61.3 ± 0.6^d^
DFR-80 + AA	57.7 ± 1.6^a^	13.9 ± 1.6^c^	71.6 ± 1.7^a^	54.5 ± 0.4^f^
DFR-60 + AW	40.8 ± 4.5^b^	17.1 ± 2.5^bc^	57.9 ± 4.0^bc^	64.2 ± 0.6^bc^
DFR-80 + AW	41.0 ± 4.0^b^	27.8 ± 3.2^a^	68.8 ± 4.1^ab^	59.2 ± 0.6^e^
P. eryngii				
DFR-00	60.3 ± 3.4^a^	5.6 ± 1.9^c^	65.8 ± 2.2^a^	70.4 ± 0.2^a^
DFR-60	29.5 ± 4.5^cb^	14.8 ± 2.1^abc^	44.3 ± 3.8^bc^	62.4 ± 0.5^b^
DFR-80	26.4 ± 3.6^c^	10.0 ± 3.4^bc^	36.5 ± 1.7^c^	57.0 ± 0.4^d^
DFR-60 + AA	29.1 ± 2.0^bc^	24.4 ± 2.2^a^	53.5 ± 3.1^ab^	56.4 ± 0.8^d^
DFR-80 + AA	41.4 ± 3.8^b^	15.7 ± 2.7^abc^	57.1 ± 3.8^ab^	51.2 ± 0.6^f^
DFR-60 + AW	34.6 ± 2.1^bc^	12.3 ± 2.9^bc^	46.9 ± 1.9^bc^	60.2 ± 0.6^c^
DFR-80 + AW	38.9 ± 2.8^bc^	18.5 ± 3.6^ab^	57.4 ± 5.9^ab^	53.5 ± 0.6^e^

a BE mean ± SE (*n* = 5) on a wet basis (normalized to 90% moisture content).

b Mean ± SE (*n* = 5) on a dry weight. Lack of letters in common indicates statistically significant differences (Tukey’s *t* test, *p* < 0.05) for comparisons of mean values between substrates.

The highest BE of the first flush of *P. eryngii* grown on fiber reject substrates was obtained from the DFR-80 + AA substrate with 41.4% (Table 4). For the other substrates, the BE ranged from approximately 26 to 39% depending on the substrate composition compared to 60.3% for the control substrate. The BE of the second flush of *P. eryngii* fruit bodies was higher in the substrates based on fiber reject, with values from approximately 10 to 24% compared to 5.6% for the control substrate. The substrates based on fiber reject that led to the highest total BE of *P. eryngii* were DFR-80 + AA (57.1%) and DFR-80 + AW (57.4%) compared to 65.8% for the control (DFR-00).

For both fungi species, fiber reject-based substrates were more degraded than the controls, and the degradation of the DFR-80 + AA substrates was larger (Table 4). This is probably due to the differences in the cultivation times (Table 3) and factors such as mycelium growth (density) and BE. The control substrates were less degraded than the others probably due to the bigger wood particle size compared to cellulose fiber as well as differences in lignin contents and other factors that affected the mycelium growth.

### 3.3 Mushroom Fruit Body Quality, Nutritional Values, and Content of Potentially Toxic Heavy Metals

Only the fruit bodies collected from the first flush were analyzed. The amount of fruit collected from the second flush did not allow for reliable analysis. The contents of ash and nutritional values are shown in Table 5.

**Table 5:** Ash Content and Nutritional Analysis of the First Flush of *P. ostreatus* and *P. eryngii* Fruit Bodies

	values given in wt % dry weight^a^			
	ash	crude fiber	crude fat	crude protein
reference values from USDA database^27^	9.3 ± 1.0	21.2 ± 3.4	3.8 ± 0.6	30.6 ± 2.9
P. ostreatus grown on diverse types of substrates^28^	6.2–9.9	12.5–24.3	1.6–3.5	14.6–31.4
P. ostreatus grown on raw fiber rejects^29^	7.1–7.1	8.1–9.0	3.0–3.5	22.3–23.7
P. ostreatus grown on birch sawdust^29^	6.4	6.4	1.0	21.0
P. ostreatus				
FR00	4.77 ± 0.03^b^	5.53 ± 0.17^a^	1.55 ± 0.18^ab^	15.8 ± 0.2^a^
FR60	5.03 ± 0.12^ab^	4.53 ± 0.15^b^	1.66 ± 0.18^ab^	11.8 ± 0.3^b^
FR80	4.87 ± 0.03^b^	4.51 ± 0.26^b^	0.92 ± 0.09^b^	11.9 ± 0.4^b^
FR60 + AA	5.17 ± 0.19^ab^	4.45 ± 0.17^b^	1.27 ± 0.06^ab^	15.0 ± 0.7^a^
FR80 + AA	4.83 ± 0.03^b^	5.18 ± 0.33^ab^	1.64 ± 0.21^ab^	14.2 ± 0.4^ab^
FR60 + AW	5.30 ± 0.06^ab^	4.36 ± 0.09^b^	1.81 ± 0.22^a^	13.9 ± 0.3^ab^
FR80 + AW	5.57 ± 0.38^a^	4.35 ± 0.08^b^	1.62 ± 0.20^ab^	14.8 ± 1.1^a^
P. eryngii				
FR00	4.13 ± 0.07^c^	4.59 ± 0.13^a^	1.71 ± 0.19^a^	9.8 ± 0.3^b^
FR60	5.67 ± 0.13^ab^	4.29 ± 0.15^a^	1.55 ± 0.12^a^	11.9 ± 0.5^ab^
FR80	5.60 ± 0.15^ab^	4.33 ± 0.18^a^	1.73 ± 0.15^a^	10.3 ± 0.5^ab^
FR60 + AA	5.33 ± 0.09^b^	4.30 ± 0.07^a^	1.65 ± 0.07^a^	11.8 ± 0.9^ab^
FR80 + AA	6.00 ± 0.01^a^	4.57 ± 0.17^a^	1.93 ± 0.29^a^	11.1 ± 0.3^ab^
FR60 + AW	5.20 ± 0.26^b^	4.28 ± 0.27^a^	2.05 ± 0.03^a^	10.7 ± 0.3^ab^
FR80 + AW	5.67 ± 0.13^ab^	4.87 ± 0.28^a^	1.72 ± 0.04^a^	17.7 ± 0.5^a^

a Mean ± SE, *n* = 3. Lack of letters in common indicates statistically significant differences (Tukey’s *t* test, *p* < 0.05) for comparisons of mean values between substrates.

Compared to the control substrate, DFR-based substrates led to fruit bodies with slightly higher contents of ash and similar contents of crude fiber, crude fat, and crude protein. The values of these quality parameters are in the range of the those for *Pleurotus* spp. mushrooms grown on similar types of substrates (Table 5). The variations are probably due to different environmental growing conditions, mushroom strains, and substrate formulation, among other factors that can affect the quality of the fruit bodies.

Fungi are known to absorb and bioaccumulate metals in the fruit bodies, one of the reasons there is interest in mycoremediation. The correct term for them is not heavy metals but potentially toxic elements, and the main route of exposure to them is through the diet. The most common toxic elements found in foodstuffs are arsenic (As), lead (Pb), cadmium (Cd), and mercury (Hg).^30^ These elements have no beneficial effects on humans and the first question when thinking about them is what the safe limits are. The Food and Drug Administration (FDA) does not establish any limits for heavy metals in foodstuffs. However, the European Commission (EC) regulation (2015/1006) set reference up-limit values given in Table 6.

**Table 6:** Contents of Potentially Toxic Elements in the First Flush of *P. ostreatus* and *P. eryngii* Fruit Bodies

	values given in μg/kg wet weight^a^			
	As	Pb	Cd	Hg
up-limit value set in the EC regulation (2015/1006)^31^	0.30 μg/day per kg of human body	300 μg/kg mushroom (ww.)	200 μg/kg mushroom (ww.)	300 μg/kg fish (ww.)
P. ostreatus grown on raw fiber rejects^29^	48–53	7.7–10.1	19.0–20.5	5.0–6.2
P. ostreatus grown on birch sawdust^29^	6.4	4.7	68	3.1
P. ostreatus				
FR00	5.00 ± 0.00^b^^d^	53.00 ± 5.13^a^	65.67 ± 4.41^a^	2.00 ± 0.00^b^^c^
FR60	24.33 ± 0.67^b^	39.67 ± 2.91^b^	31.67 ± 0.88^b^	4.67 ± 0.37^b^
FR80	23.33 ± 0.67^b^	16.67 ± 2.91^c^	31.33 ± 3.53^b^	4.93 ± 0.13^ab^
FR60 + AA	17.67 ± 1.20^c^	28.67 ± 1.76^bc^	32.67 ± 2.91^b^	3.63 ± 0.15^b^
FR80 + AA	14.00 ± 0.58^c^	37.67 ± 3.48^b^	24.00 ± 1.53^b^	3.90 ± 0.35^b^
FR60 + AW	34.00 ± 1.76^a^	22.33 ± 1.20^c^	25.33 ± 3.18^b^	4.93 ± 0.22^ab^
FR80 + AW	34.67 ± 1.15^a^	21.33 ± 1.20^c^	22.33 ± 0.88^b^	6.07 ± 0.47^a^
P. eryngii				
FR00	5.00 ± 0.00^b^^a^	24.33 ± 2.91^a^	49.00 ± 1.73^a^	2.00 ± 0.00^b^^e^
FR60	6.23 ± 1.23^a^	9.03 ± 0.62^b^	34.33 ± 5.70^b^	4.40 ± 0.55^bcd^
FR80	6.10 ± 0.15^a^	15.00 ± 1.53^ab^	28.67 ± 4.91^b^	6.00 ± 0.51^ab^
FR60 + AA	5.00 ± 0.00^b^^a^	19.00 ± 3.21^ab^	26.67 ± 2.73^b^	3.70 ± 0.10^d^
FR80 + AA	5.00 ± 0.00^b^^a^	16.00 ± 3.06^ab^	31.67 ± 1.20^b^	6.33 ± 0.41^a^
FR60 + AW	5.07 ± 0.07^a^	20.67 ± 2.40^ab^	20.00 ± 0.58^b^	4.00 ± 0.36^cd^
FR80 + AW	6.60 ± 0.56^a^	20.00 ± 2.65^ab^	20.00 ± 1.00^b^	5.60 ± 0.38^abc^

a Mean ± SE normalized to 90% moisture content, *n* = 3.

b Below detection limits. Lack of letters in common indicates statistically significant differences (Tukey’s *t* test, *p* < 0.05) for comparisons of treatment means between substrates.

The results showed that *P. ostreatus* has a better bioaccumulation capacity for As than *P. eryngii* (Table 6). The levels of As in the *P. eryngii* fruit bodies were in most cases below the detection limit (5 μg/kg). The recommended up-limit value given in EC regulations for As is 0.3 μg/day per kg of human body. Taking the fruit bodies with the higher As content, that is, those grown in the FR80 + AW substrate, a person of 60 kg could ingest 0.52 kg of *P. ostreatus* or 2.72 kg of *P. eryngii* per day without surpassing the recommended level. The contents of Pb and Cd in the *P. ostreatus* and *P. eryngii* fruit bodies grown on substrates containing DFR were lower than that of the fruit bodies from the control substrate (DFR-00), and their concentrations were well below the limit values given in EC regulations. The content of Hg in the *P. ostreatus* and *P. eryngii* fruit bodies grown in DFR substrates was two to three times higher than that of the fruit bodies from the control substrates with a value below the detection limit (2 μg/kg). However, the concentration of Hg was, in all cases, well below the up-limit values recommended in EC regulations.

### 3.4 Characterization of Activated Biochar from Fiber Reject Spent Substrates

The substrate based on fiber rejects that led to the highest total BE (FR80 + AA where *P. ostreatus* was cultivated) was chosen as raw material for the production of activated biochar using H_3_PO_4_ and KOH as activating agents.

#### 3.4.1 Textural Properties of the Activated Carbons

**Figure 2:**
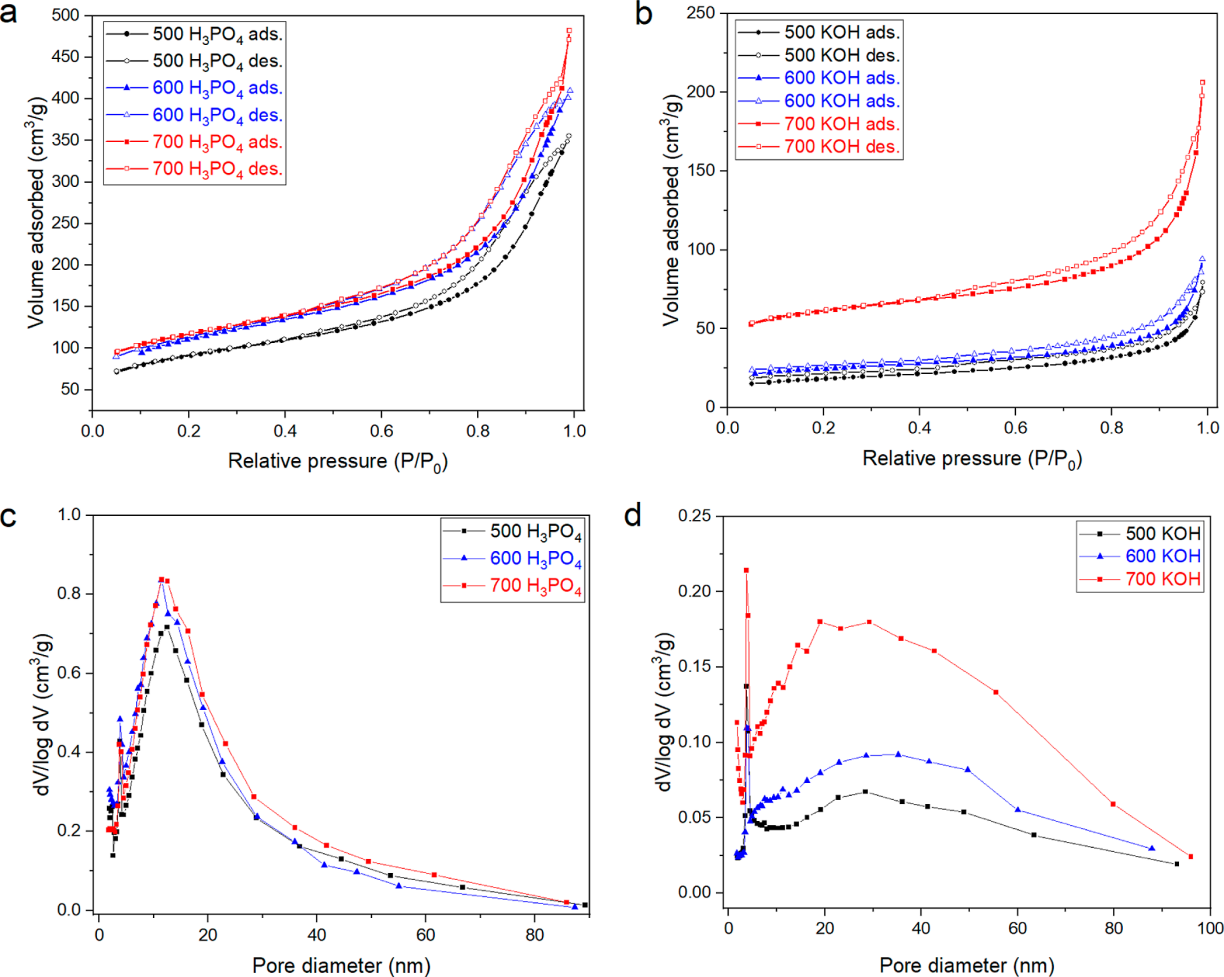
N_2_ adsorption/desorption isotherms (a,b) and pore size distribution (c,d) for the activated carbons developed from FR80 + AA SMS. Solid lines are only to guide the eyes.

All activated biochars used for the experiments had a particle size between 80 and 100 μm. Figure 2 shows the nitrogen adsorption/desorption isotherms and pore size distributions. The textural characteristics derived from the isotherms are listed in Table 7. According to the IUPAC classification, the isotherms showed intermediate features between type II, III, and IV depending on the carbon, which means that mesopores and macropores account for most of the total surface area. Increasing the pyrolysis temperature led to an increase in the amounts of N_2_ adsorbed, and a hysteresis loop indicates capillary condensation on the external surface of mesopores.^32,33^

**Table 7:** Textural Properties of the Activated Carbons

	H_3_PO_4_			KOH		
parameters	500 °C	600 °C	700 °C	500 °C	600 °C	700 °C
*S*_BET_(m^2^/g)	302	376	396	61	81	199
external surface area (m^2^/g)	247	279	282	41	43	95
mesopore area (%)	81.8	74.3	71.1	66.9	53.6	47.8
micropore area (m^2^/g)	55	96	115	20	38	104
micropore area (%)	18.2	25.7	28.9	33.1	46.4	52.1
total pore volume (cm^3^/g)	0.550	0.634	0.745	0.123	0.146	0.319
micropore volume (cm^3^/g)	0.032	0.040	0.057	0.010	0.019	0.053
micropore volume (%)	5.80	6.25	7.71	8.31	13.2	16.6
mesopore volume (cm^3^/g)	0.518	0.594	0.688	0.113	0.127	0.266
mesopore volume (%)	94.2	93.7	92.3	91.7	86.8	83.4

The pore size distribution (Figure 2c,d) of the activated biochars produced with the same activation agent showed a similar pattern with the majority of the pores between 2 and 80 nm. For both activation agents, the total surface area (*S*_BET_), total pore volume, and micropore area increased with the increase of the pyrolysis temperature (Table 7).

**Figure 3:**
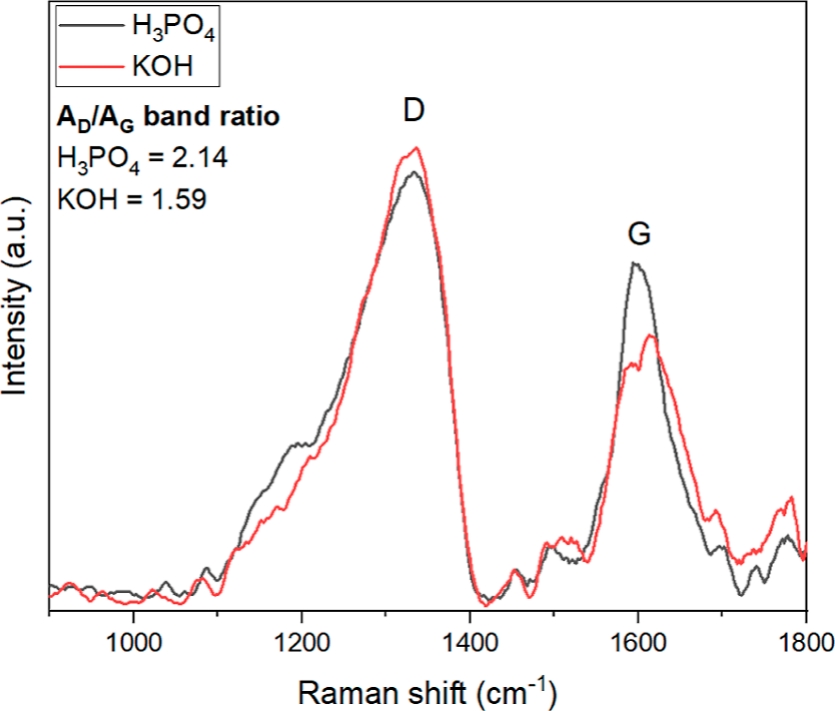
Raman spectra of the activated biochars produced at 700 °C and the area ratios of the D-band peak to the G-band peak.

Raman spectroscopic analysis (Figure 3) was carried out on the activated biochars with the highest surface area, that is, those pyrolyzed at 700 °C. This method is among the most informative for the evaluation of the degree of graphitization and structural perfection of the carbon matrix.

Figure 3 shows the D and G bands for each activated biochar. The integrated area under each band peak was calculated using OriginPro (2020) software. The area ratios of the D-band peak to the G-band peak were 2.14 for the activated biochar produced with H_3_PO_4_ and 1.59 for the one produced with KOH, meaning that the H_3_PO_4_ carbon has more disordered graphite structures, that is, higher amount of structural defects such as grain boundaries, bonding disorders, and vacancies. As a comparison, the *A*_D_/*A*_G_ ratio of pure graphite is 0.04.^34^ A high degree of structural defects may affect the interaction between the adsorbate and adsorbent and lead to a better adsorption performance.

#### 3.4.2 Influence of the Activated Biochar Dose on the Removal of Acetaminophen and Amoxicillin from Aqueous Solution

**Figure 4:**
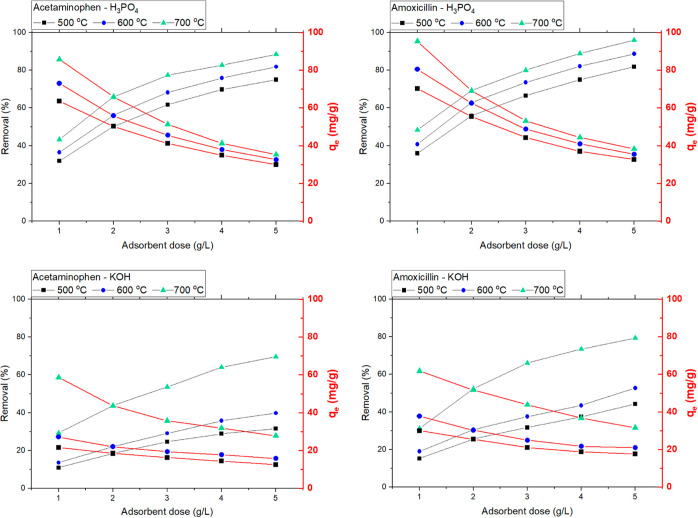
Influence of the adsorbent dose on the percentage of removal and adsorption capacity of acetaminophen and amoxicillin at equilibrium. Initial drug concentration of 200 mg/L, pH 6.0, temperature of 22 °C, and contact time of 6 h. Solid lines are only to guide the eyes.

The adsorbent dosage measurements for 200 mg/L acetaminophen and amoxicillin solutions are shown in Figure 4.

At a constant initial drug concentration, increasing the adsorbent dose provides a larger number of adsorption sites and hence the enhancement of the drug percentage of removal but reduced adsorption capacity (*q*_e_). Comparing the dosage curves (Figure 4) with the surface area analysis of the activated biochars (Table 7), one can see that the highest efficiencies were obtained with the samples produced at 700 °C, that is, those with the highest surface area. For further characterization of the adsorbents, only these activated biochars were used. The adsorbent dosage was set to 2 g/L for both samples and was chosen based on the variation of the adsorption capacity (*q*_e_), that is, the differences between 1 and 2 g/L are slightly higher than that between 2 and 3 g/L. Apart from this, using an adsorbent dosage of 2 g/L reduces the generation of waste and simplifies the experimental work.

#### 3.4.3 Influence of the pH on the Adsorption Capacity of Acetaminophen and Amoxicillin

The influence of the pH of the solution of contaminants is one factor that may affect the adsorption process. The structural formula and pKa values of each drug are shown in Supporting Figure S2. The optimum pH depends on the chemical nature of the adsorbent, as well as the solubility and structure of the adsorbate and that pH.^35^

**Figure 5:**
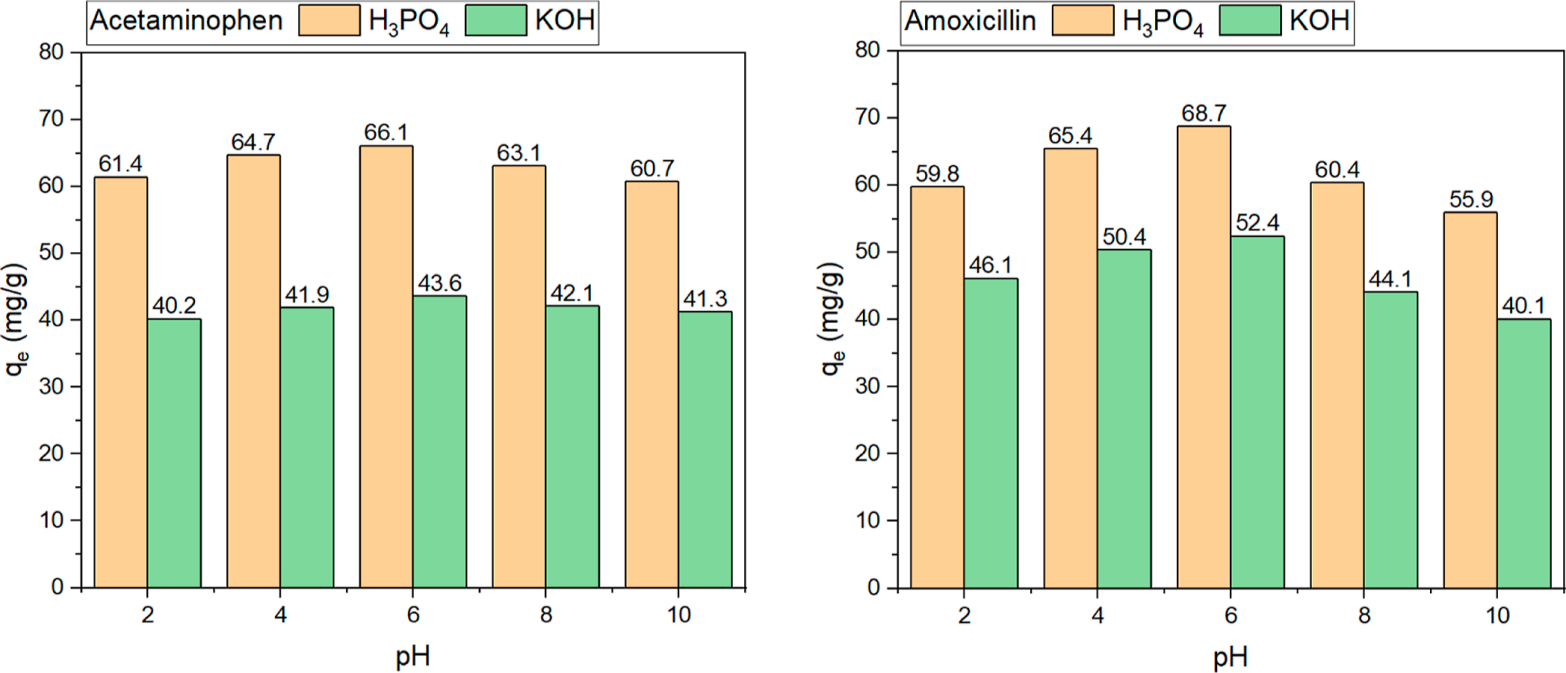
Influence of the pH on the adsorption capacity. Initial drug concentration of 200 mg/L, adsorbent dose of 2 g/L, temperature of 22 °C, and contact time of 6 h for activated biochars produced at 700 °C.

According to the results shown in Figure 5, the highest *q*_e_ for acetaminophen and amoxicillin for both activated biochars was obtained at a slightly acidic pH. The differences between q_e_ at acidic and basic pH were slightly higher for amoxicillin. Perhaps these differences are related to the acid dissociation constant (pK_a_) values of the adsorbates that may have influenced their sorption capacities under the studied range of pHs.^36−40^ Acetaminophen is a weak acid having a pK_a_ at around 9.46, while amoxicillin has four pK_a_ values, including a strong acid part with a pK_a_ value of 3.23 (see Supporting Figure S2b); both molecules exist in their molecular forms at pH lower than its pK_a_ values while at higher pHs, they exist in its ionic forms. Thus, amoxicillin remains with an ionic part at a wider pH range, which could reflect its higher adsorption capacity compared to acetaminophen.

It is proven that the electrostatic mechanism does not exert a remarkable influence on the overall sorption mechanism for both drugs. Other studies that studied the adsorption of acetaminophen^41^ and amoxicillin^42^ showed similar results to what was obtained in this work. Further adsorption studies were carried out at the pH that gave the best results (pH 6).

#### 3.4.4 Kinetic of Adsorption

**Figure 6:**
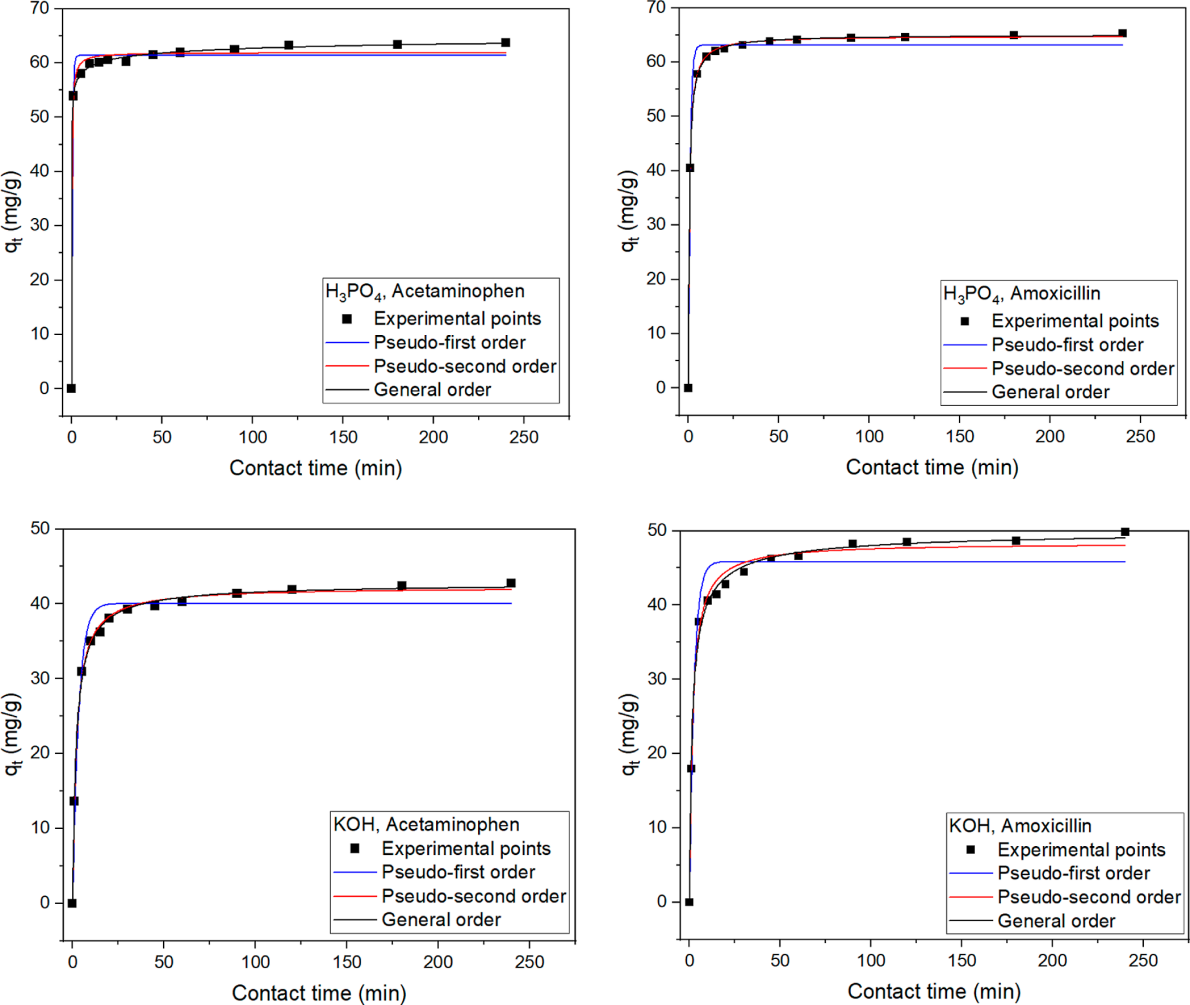
Kinetic data for adsorption of acetaminophen and amoxicillin onto the activated biochars produced at 700 °C and comparison between experimental measurements and predictions of the models. Initial drug concentration of 200 mg/L, pH 6.0, temperature of 22 °C, and adsorbent dose of 2 g/L.

The kinetic measurements were fitted using the nonlinear pseudo-first-order, pseudo-second-order, and general-order models.^43,44^ Equations are given in the Supporting Information (eqs S1–S3). The results from the measurements are shown in Figure 6.

The adsorption increased sharply at contact times of less than 10 min and slowed gradually as equilibrium was approached. For both activated biochars, equilibrium was attained between 20 min and 1 h. The fitting parameters obtained from each model are presented in Table 8. The *R*^2^_adj_ and the SD values were used to judge the fitting of the models. Low SD and *R*^2^_adj_ near 1 mean a low discrepancy between experimental and theoretical adsorption capacity (*q*).^43,44^ According to the results, the general order kinetics model showed a better fitting of the experimental measurements, that is, lower SD and higher *R*^2^_adj_, meaning that the values of q foreseen by this model are closest to the q values measured experimentally.

**Table 8:** Kinetic Parameters for Adsorption onto Carbons Produced at 700 °C

	H_3_PO_4_		KOH	
model	acetaminophen	amoxicillin	acetaminophen	amoxicillin
Pseudo-First-Order				
*q*_e_(mg/g)	61.35	63.12	40.03	45.83
*k*_1_(1/min)	2.1106	1.0073	0.3010	0.3886
*R*^2^_adj_	0.9903	0.9881	0.9703	0.9571
SD (mg/g)	1.679	1.985	2.203	2.995
Pseudo-Second-Order				
*q*_e_(mg/g)	61.89	64.79	42.25	48.36
*k*_2_(g/mg min)	0.01751	0.02552	0.01151	0.01292
*R*^2^_adj_	0.9946	0.9997	0.9977	0.9923
SD (mg/g)	1.245	0.2891	0.6092	1.269
General Order				
*q*_e_(mg/g)	65.58	68.07	42.81	50.35
*k*_N_[(g/mg)^*n*−1^/min]	0.01311	0.01751	0.006311	0.001741
*N*	2.132	2.104	2.176	2.529
*h*_o_(mg/g min)	97.11	125.8	22.37	35.07
*R*^2^_adj_	0.9995	0.9998	0.9979	0.9947
SD (mg/g)	0.3731	0.2362	0.5811	1.049

The order of adsorption rate (n) in the general order kinetic model changes depending on the type of adsorbents and adsorbates, which complicates the comparison of kinetic parameters. Therefore, the initial adsorption rate h_o_ (eq 4) is useful to evaluate the results given by this model.^45^
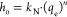
4where *h*_o_ denotes the initial sorption rate (mg/g min), *k*_N_ is the rate constant [(g/mg)^*n*−1^/min], *q*_e_ is the amount adsorbed at equilibrium (mg/g), and *n* is the order of the kinetic model.

Comparing the *h*_o_ values for the general order model (Table 8), one can see that the adsorption of amoxicillin, that is, the removal of the drug from the solution, was faster. The activated carbon made with H_3_PO_4_ as an activation agent resulted in higher h_o_ values; however, this is not surprising considering the larger surface area of this carbon.

#### 3.4.5 Equilibrium Adsorption Isotherms

Adsorption isotherms were used to describe the relationship between the amount of the adsorbate adsorbed by the adsorbent at equilibrium (*q*_e_) and the concentration of the adsorbate that remained in the solution at equilibrium (*C*_e_). The adsorption parameters obtained from equilibrium models provide an idea of the adsorption mechanisms, the affinity of the adsorbent for the adsorbate, and the maximum adsorption capacity (*q*_max_) of the adsorbent. The results from equilibrium measurements were fitted using the nonlinear Langmuir, Freundlich, and Sips models.^43,44^ Equations are given in the Supporting Information (eqs S4–S6).

**Figure 7:**
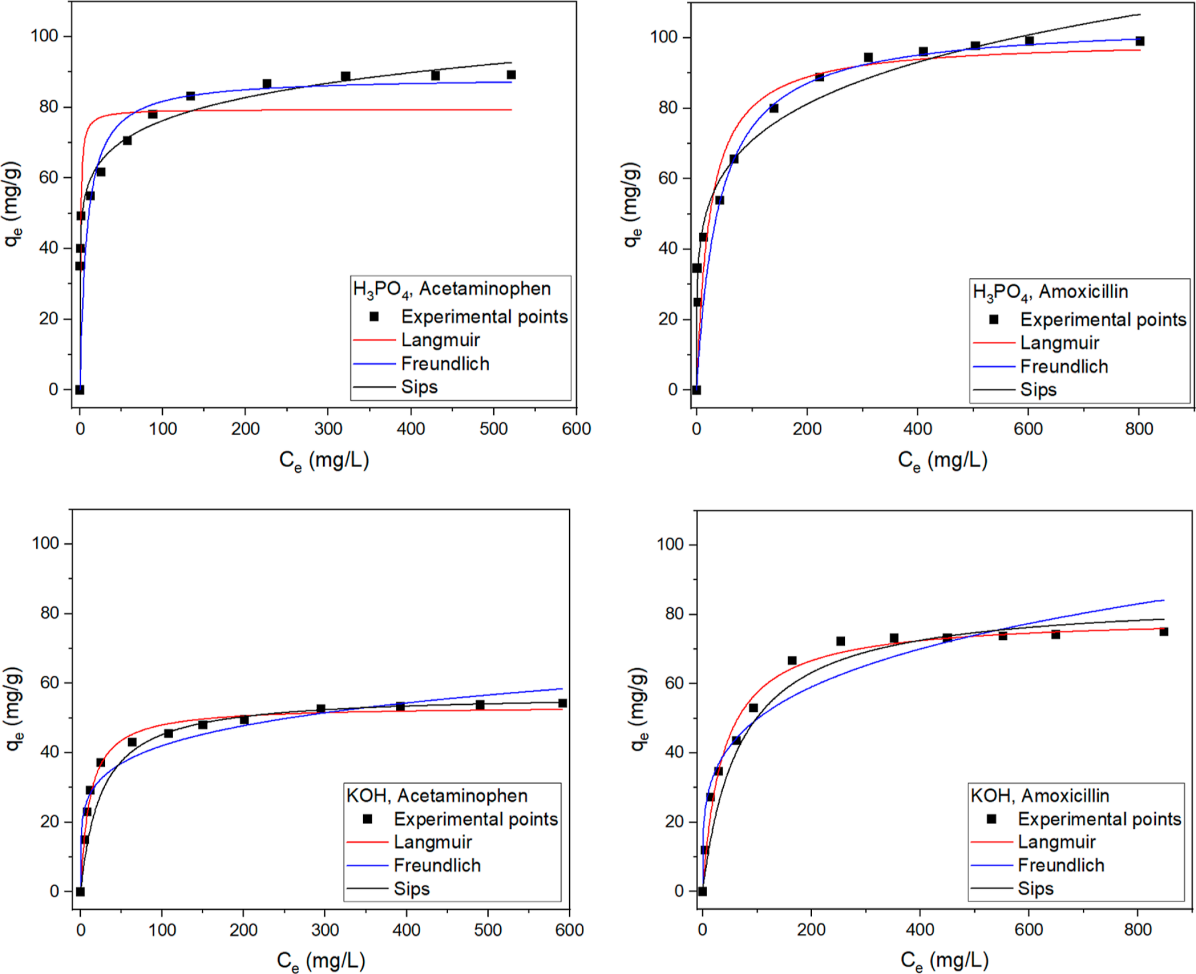
Isotherms of adsorption of acetaminophen and amoxicillin onto activated carbons produced at 700 °C. Experimental conditions: pH 6.0, temperature 22 °C, adsorbent dose 2 g/L, and contact time 2 h.

The isotherms of adsorption (Figure 7) were carried out at a temperature of 22 °C using the optimal pH conditions, as described in Section 3.4.3, and a contact time of 2 h to make sure that the adsorption process reaches equilibrium. The parameters obtained from the nonlinear regression of each model are shown in Table 9.

**Table 9:** Isotherm Parameters for Adsorption of Acetaminophen and Amoxicillin onto Activated Carbos Produced at 700 °C

	H_3_PO_4_		KOH	
model	acetaminophen	amoxicillin	acetaminophen	amoxicillin
Langmuir				
*q*_max_(mg/g)	79.38	99.39	53.41	79.23
*K*_L_(L/min)	1.381	0.04211	0.08811	0.02642
*R*^2^_adj_	0.7081	0.8661	0.9892	0.9871
SD (mg/g)	16.61	12.15	1.721	2.917
Freundlich				
*K*_F_((mg/g) (mg/L)^−1/*n*^_F_)	44.28	28.71	17.93	16.08
*n*_F_	7.471	5.093	5.409	4.074
*R*^2^_adj_	0.8961	0.9712	0.8431	0.8322
SD (mg/g)	6.071	5.491	4.069	6.835
Sips				
*q*_max_(mg/g)	88.45	104.6	56.81	84.97
*K*_S_(L/mg)	0.1211	0.0252	0.0391	0.0145
*n*_S_	1	1	1	1
*R*^2^_adj_	0.7261	0.8192	0.9331	0.9652
SD (mg/g)	9.832	14.93	3.251	4.463

The suitability of each model was judged using the *R*^*2*^_Adj_ and SD values.^43,44^ Regardless of the type of drug, isotherms of adsorption onto the activated biochar produced with H_3_PO_4_ were better described by the Freundlich model, and isotherms of adsorption onto the activated biochar produced with KOH were better described by the Langmuir model.

The good agreement between the Freundlich model with the experimental data for the carbon produced with H_3_PO_4_ points to multilayer adsorption with heterogeneity in the energy of the adsorption sites. On the other hand, the good agreement between the Langmuir model with the experimental data for the carbon produced with KOH points to a process dominated by monolayer adsorption on the surface of the activated carbon particles as well as a finite number of energetically equivalent adsorption sites.

Analyzing the maximum adsorption capacities (*q*_max_) values obtained from the Langmuir or Sips isotherms, one can see that both carbons resulted in higher removal of amoxicillin (Table 9). This was also observed in the kinetic measurements. Due to the dimensions of these molecules, that is, 1.361 nm length for amoxicillin and 0.887 nm length for acetaminophen,^35^ it is expected that they can access wide and narrow pores on the adsorbent surface (Figure 2). Apart from this, there are big differences in the polar surface area of the adsorbates, that is, 16.27 nm^2^ for amoxicillin and 4.93 nm^2^ for acetaminophen.^35^ A molecule with a larger polar surface area means stronger charge-based electrostatic interactions, which may explain why the removal of amoxicillin was higher than acetaminophen. Saucier *et al.*(35) found similar results when studying the adsorption of these two drugs onto magnetic activated carbons; the removal of amoxicillin was higher compared to acetaminophen, which is in agreement with the results obtained in this work.

#### 3.4.6 Mechanisms of Adsorption

It was found that the removal of acetaminophen and amoxicillin from aqueous solutions is related to the surface area of the activated biochars, meaning that the pore-filling mechanism played an important role in the process. Measurement of the adsorption capacity (*q*_e_) at different pH showed that the electrostatic mechanism does not exert a remarkable influence on the overall sorption mechanism of both drugs.^46−49^ However, interactions such as van der Waals (hydrophobic interactions and π–π stacking), hydrogen bonds, and polar interactions of the oxygen and nitrogen groups of the drugs with the polar groups of the biochars took place. A diagrammatic scheme of the adsorption mechanism is shown in Supporting Figure S3.

## 4 Conclusions

Cellulose fiber rejects from industrial-scale recycling of waste papers were processed using a cyclone dryer followed by a sieving process that together allowed for combined drying and removal of contaminants such as ash and microplastics. The upgraded cellulose fiber rejects (DFR) was used in substrates for the cultivation of *P. ostreatus* and *P. eryngii* mushrooms. Substrates containing 60 and 80 wt % DFR were tested. The pH of these two substrates was adjusted to a suitable pH for mycelium growth by using AA and AW, and the effect that this correction had on the BE and quality parameters of the fruit bodies was measured. A standard substrate formula based on birch wood was used as a control substrate. The cellulose fiber reject-based spent mushroom substrate (SMS) was used for the production of activated biochar using H_3_PO_4_ and KOH as chemical activators. Acetaminophen and amoxicillin were used as model micropollutants to test the activated carbons. The main results from this research showed that:

- By using a feeding rate of approximately 250 kg/h and a drying air temperature of 70 °C, the initial moisture content of the raw fiber rejects (57.8 wt %) was reduced to 5.4 wt %, and the ash content (39.2 wt %) was reduced to 21.5 wt %. Consequently, the content of potentially toxic heavy metals was reduced substantially.
- The mycelium growth in de-ashed fiber reject (DFR) substrates was faster compared to that of the control substrates.
- The correction of the pH of the DFR-60 (7.10) and DFR-80 (7.73) substrates to a suitable pH for the cultivation of white-rot fungi (approximately 6.5) by adding AA led to longer colonization times and higher mycelium density compared to the other substrates.
- The addition of AA to the DFR-80 substrate delayed the harvest of the first and second flush of *P. ostreatus* and *P. eryngii* fruit bodies but led to a higher or comparable total BE compared to that of the control substrate.
- *P. ostreatus* and *P. eryngii* fruit bodies’ contents of ash, crude fiber, crude fat, and crude protein were comparable to that of those grown on the control substrates.
- The contents of potentially toxic heavy metals, that is, arsenic (As), lead (Pb), cadmium (Cd), and mercury (Hg) in *P. ostreatus* and *P. eryngii* fruit bodies were lower than reference values for food products set in EC regulations.
- SMS from cultivation *P. ostreatus* on the substrate with 80 wt % cellulose fiber reject with the addition of AA (DFR-80 + AA) was used as a carbon precursor. The maximum surface areas were obtained for samples pyrolyzed at 700 °C, that is, 396 m^2^/g (for the H_3_PO_4_-activated biochar) and 199 m^2^/g (for the KOH-activated biochar).
- The kinetics of adsorption of acetaminophen and amoxicillin for both activated biochars were fitted to the general order model.
- Irrespective of the type of drug, the equilibrium data of adsorption onto H_3_PO_4_-activated biochar was well fitted by the Freundlich isotherm, and adsorption onto KOH-activated biochar was better described by the Langmuir isotherm.
- The adsorption of acetaminophen and amoxicillin onto the activated biochars is governed by the pore-filling mechanism.

## Supporting Information Available

Photograph of the cyclone dryer experimental setup; structural formula of acetaminophen and amoxicillin; diagrammatic scheme of the adsorption mechanism; kinetic of adsorption models; and equilibrium of adsorption isotherms (PDF)
