# Supplementary material for: Cellulose Fiber Rejects as Raw Material for Integrated Production of Pleurotus spp. Mushrooms and Activated Biochar for Removal of Emerging Pollutants from Aqueous Media
Source: ACS Omega. 2023 Jan 31;8(6):5361–76. doi: 10.1021/acsomega.2c06453 (PMC9933083; doi:10.1021/acsomega.2c06453)
Supplement: Supplementary file 1 — ao2c06453_si_001.pdf [file ao2c06453_si_001.pdf]

Supporting information

Cellulose fibre rejects as raw material for integrated production of *Pleurotus* spp. mushrooms and activated biochar for removal of emerging pollutants from aqueous media

Alejandro Grimm[1,*], Feng Chen [1], Glaydson Simões dos Reis[1], Van Minh Dinh[2], Santosh Govind Khokarale[2], Michael Finell[1], Jyri-Pekka Mikkola[2,3], Malin Hultberg[4], Guilherme L. Dotto[5], Shaojun Xiong[1]

[1] Department of Forest Biomaterials and Technology, Swedish University of Agricultural Sciences, SE-901 83, Umeå, Sweden

[2] Technical Chemistry, Department of Chemistry, Chemical-Biological Centre, Umeå University, SE-901 87, Umeå, Sweden

[3] Industrial Chemistry and Reaction Engineering, Johan Gadolin Process Chemistry Centre, Åbo Akademi University, FI-20500, Åbo-Turku, Finland

[4] Department of Biosystems and Technology, Swedish University of Agricultural Sciences, SE-230 53, Alnarp, Sweden

[5] Research Group on Adsorptive and Catalytic Process Engineering (ENGEPAC), Federal University of Santa Maria, Av. Roraima, 1000-7, BR 97105-900, RS, Santa Maria, Brazil

* Corresponding author.

E-mail address: Alejandro.Grimm@slu.se

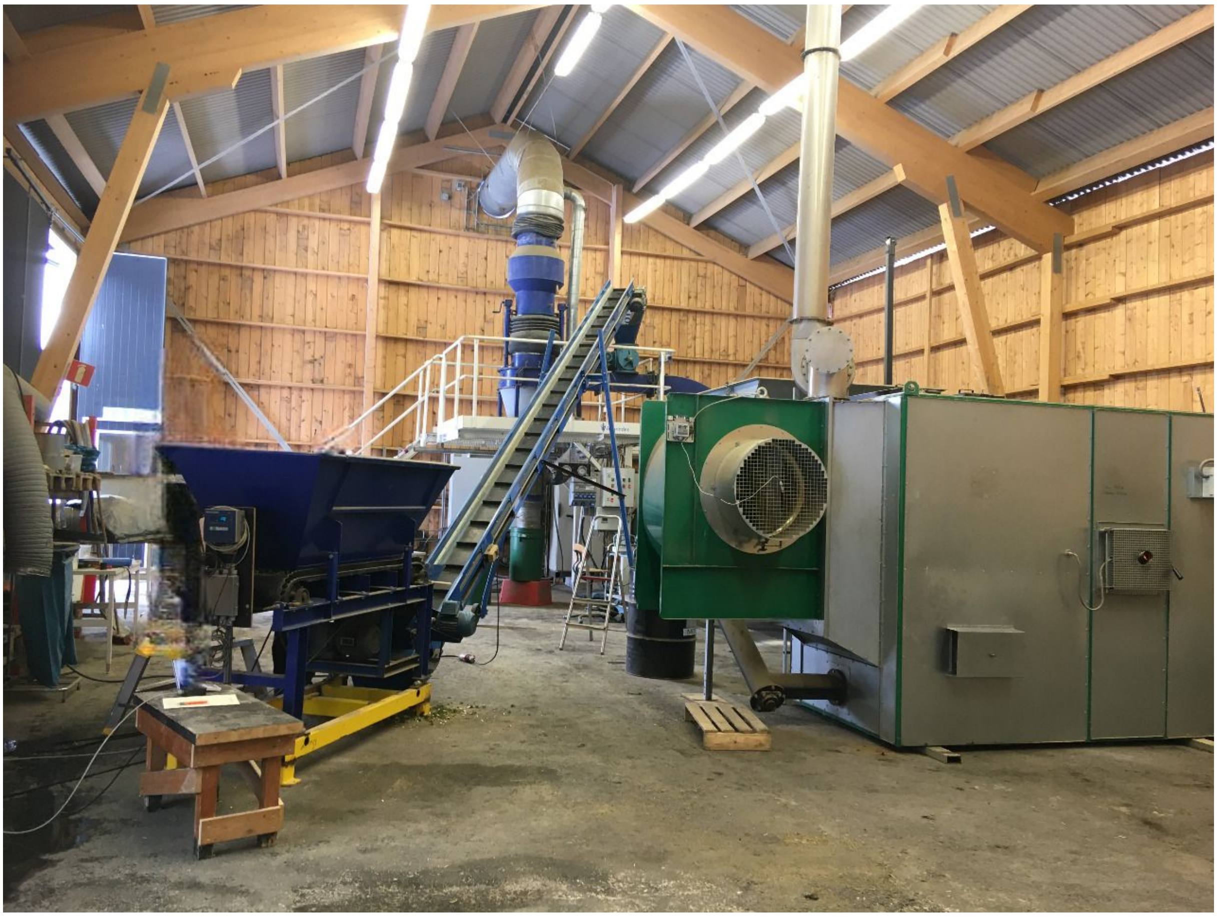

Figure S1. Photograph of the cyclone dryer experimental setup.

a) Acetaminophen

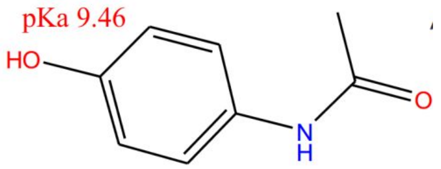

b) Amoxicillin

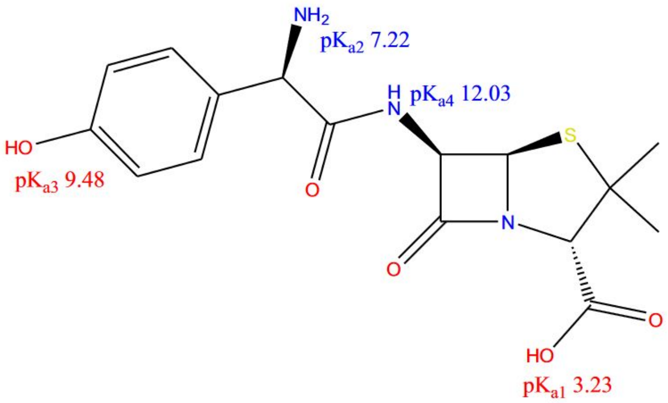

Figure S2. Structural formula of acetaminophen and amoxicillin. pKa values are indicated in the figures.

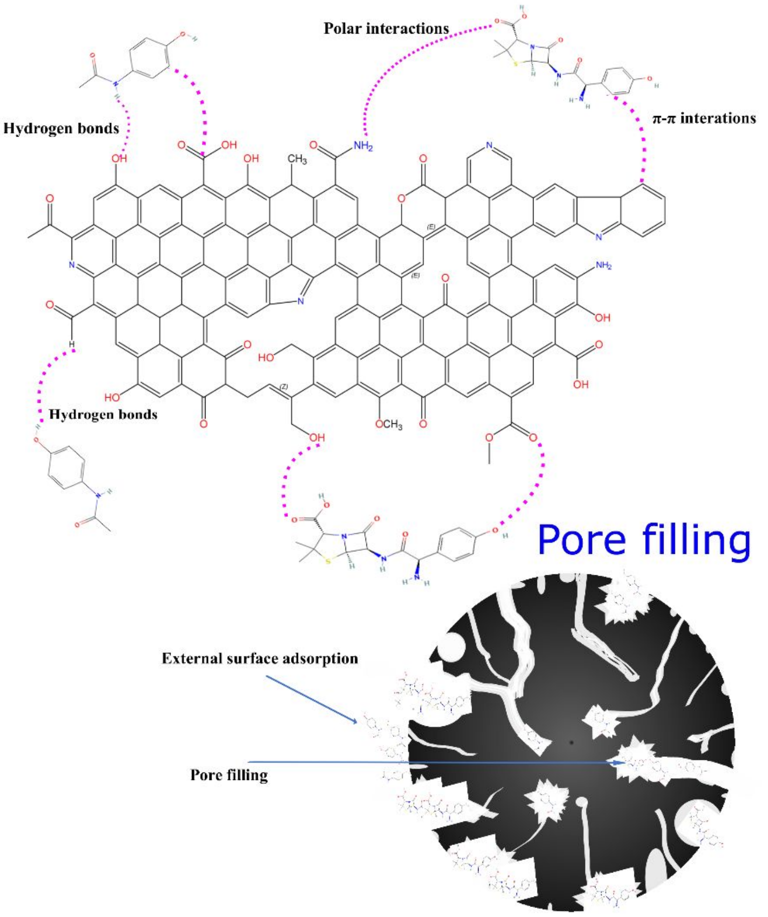

Figure S3. Diagrammatic scheme of the adsorption mechanism

Kinetic of adsorption models

Pseudo-first-order: $q_t = q_e\,(1 - \exp^{-k_1.t})$ S1

pseudo-second-order: $q_t = \frac{k_2.q_e^2.t}{1 + q_e.k_2.t}$ S2

General order: $q_t = \left(q_e - \frac{q_e}{[k_N.(q_e)^{n-1}.t.(n-1)+1]^{1/(1-n)}}\right)$ S3

where, t denotes the contact time (min); q_t, q_e are the adsorption capacities at time t and at equilibrium, respectively (mg/g); k_1 is the pseudo-first-order rate constant (L/min); k_2 is the pseudo-second-order rate constant (g/mg min); k_N is the general-order constant rate $[(g/mg)^{n-1})/min]$, and **n** is the dimensionless general-order adsorption rate.

Equilibrium of adsorption isotherms

Langmuir: $q_e = \frac{q_{max}.K_L.C_e}{1 + K_L.C_e}$ S4

Freundlich: $q_e = K_F.C_e^{1/n_F}$ S5

Sips: $q_e = \frac{q_{max}.K_S.C_e^{1/n_S}}{1 + K_S.C_e^{1/n_S}}$ S6

where, $\mathbf{q_e}$ denotes the amount of adsorbate adsorbed at the equilibrium (mg/g); $\boldsymbol{C_e}$ is the adsorbate concentration at equilibrium (mg/L); $\boldsymbol{q_{max}}$ is the maximum adsorption capacity of the adsorbent (mg/g); $\mathbf{K_L}$ and $\mathbf{K_s}$ are the Langmuir and Sips equilibrium constant (L/mg), respectively; $\mathbf{K_F}$ is the Freundlich equilibrium constant [(mg/g) (mg/L)$^{-1/n_F}$]; $\mathbf{n_F}$ and $\mathbf{n_S}$ are the dimensionless exponents of the Freundlich and Sips model, respectively.
